# Spinal muscular atrophy type I in a 3.5-month-old male infant: A case report

**DOI:** 10.1097/MD.0000000000048512

**Published:** 2026-04-24

**Authors:** Raja Nasir Nawaz, Wafa Asjad, Roshaan Bashir, Sheikh Ali Ahmed Saeed, Muhammad Zain Tayyab, Muddassir Khalid

**Affiliations:** a Department of Paediatrics, Al-Nafees Medical College and Hospital, Islamabad, Pakistan; b Department of Paediatrics, Nishtar Medical University, Multan, Pakistan.

**Keywords:** case report, floppy infant, hypotonia, motor neuron disease, spinal muscular atrophy

## Abstract

**Rationale:**

Spinal muscular atrophy (SMA) is a rare autosomal recessive neuromuscular disorder that causes muscle weakness and hypotonia in infants due to survival motor neuron (SMN) protein degeneration. There are 5 recognized main subtypes of SMA, based on the age symptom onset, disease severity, and life expectancy. SMA type I (Werdnig–Hoffmann disease) is the most severe form, with symptom onset before 6 months of age.

**Patient concerns:**

We report the case of a 3.5-month-old male infant who presented with complaints of feeding difficulty, weak sucking power, reduced muscle tone, tongue fasciculations, and delayed motor milestones since birth. There was no cognitive or sensory impairment.

**Diagnoses:**

Antenatal history revealed polyhydramnios and reduced fetal movements in the third trimester. Electromyography revealed severe motor neuropathy in lower limbs, and multiplex ligation-dependent probe amplification analysis confirmed homozygous deletion of the survival motor neuron 1 gene, establishing the diagnosis of SMA type I.

**Interventions:**

The patient was managed with supportive measures, including feeding support via a nasogastric tube, respiratory monitoring, and genetic counseling for the family.

**Outcomes:**

Regular follow-up was advised. Disease-modifying therapies were discussed, but were not available due to resource limitations.

**Lessons:**

Early recognition and diagnosis of SMA Type I are important to improve survival and clinical outcomes in the patient. Genetic counseling, establishing standardized diagnostic procedures, and ensuring access to new treatments are crucial for optimizing patient care.

## 1. Introduction

Spinal muscular atrophy (SMA) refers to a group of inherited disorders characterized by progressive degeneration of anterior horn cells in the spinal cord, leading to muscular atrophy and weakness.^[1]^ The most common form, SMA type I, is an autosomal recessive disorder caused by a homozygous deletion or mutation of the survival motor neuron 1 (SMN1) gene located on chromosome 5q13.^[2]^ Clinical subtypes of SMA are further classified based on the age of onset and disease severity. Spinal muscular atrophy type I is the second most prevalent and potentially fatal autosomal recessive disorder. It affects approximately 1 in every 6000 to 8000 live births and has an asymptomatic carrier frequency of 1 in 34.^[3]^ Infants with SMA type I typically present with symptoms before 6 months of age. Clinical features include profound hypotonia with a characteristic “floppy infant” appearance, symmetrical flaccid paralysis and often poor head control.^[4]^ Other symptoms include tongue fasciculations, weak cry, feeding and swallowing difficulties, and recurrent choking episodes. Progressive respiratory distress is common and is characterized by paradoxical breathing and a bell-shaped chest appearance. Despite severe motor impairment, cognitive and sensory functions remain intact. This work has been reported in line with the CAse REport (guidelines) checklist.^[5]^

## 2. Case presentation

A 3.5-month-old male infant presented to the pediatric outpatient department with complaints of decreased spontaneous body movements and feeding difficulty since birth. According to the mother, there had been a noticeable worsening of reduced movements of both upper and lower limbs and poor feeding over the past 4 days. The sucking power was markedly reduced, and feeding took longer than usual, with frequent drooling of milk from the mouth. The parents also reported occasional choking episodes during feeding.

On systemic inquiry, there was no history of trauma, fever, seizures, head injury, loss of consciousness, vomiting, diarrhea, or skin rash. Urine output and bowel habits were normal. Antenatal history was unremarkable during the first and second trimesters, with regular antenatal follow-ups. The mother had taken multivitamins and hematinics and had no history of fever, rash, infection, or any chronic illness like gestational diabetes mellitus or pregnancy-induced hypertension during pregnancy. Tetanus vaccination was done during the second trimester. During the third trimester, reduced fetal movements and polyhydramnios were noted.

Natal history revealed a spontaneous vaginal delivery with an immediate cry. The infant had a good Appearance, Pulse, Grimace, Activity, Respiration score and a birth weight of about 2.8 kg. Developmental assessment showed absent head control, failure to push up with the arms during prone positioning, and delayed fine motor skills, including poor handgrip strength. However, social and cognitive functions appeared intact, as the infant paid attention to his mother’s face, followed moving objects with his eyes, and gave appropriate responses to voices and a social smile. Immunizations were up to date according to the Expanded Programme on Immunization schedule. There was a significant family history of a younger sibling who died at 10 months of age due to aspirational pneumonia. The parents were consanguineously related.

On physical examination, the infant was lying in the mother’s lap in a frog-leg posture with minimal spontaneous movements. Vital signs included a respiratory rate of 48 breaths per minute, a heart rate of 130 beats per minute, and a body temperature of 97 °F. Anthropometric measurements showed a length of 57 cm and a head circumference of 37 cm (below average for age). No abnormal or coarse facial features, clubbing, or cold clammy skin were observed. There were no signs of encephalopathy, although tongue fasciculations were present. The modified Glasgow Coma Scale score was 13/15. Neurological examination revealed generalized hypotonia with floppy limbs and reduced resistance to passive movements. Muscle power was graded as 2/5 in all 4 limbs. The Moro reflex was absent, and deep tendon reflexes were diminished. No goiter or other dysmorphic features were noted.

### 2.1. Investigations

Laboratory investigations showed a hemoglobin level of 12 g/dL, hematocrit of 35%, mean corpuscular volume of 76 fL, mean corpuscular hemoglobin of 26 pg, and a platelet count of 505,000/µL. Inflammatory markers, renal function tests, serum electrolytes, liver function tests, and thyroid function tests were normal. Creatine phosphokinase (CPK) levels were elevated in the blood (Table 1). Cranial ultrasonography and echocardiography were normal, and chest radiography showed clear lung fields. To evaluate a possible neuropathic or anterior horn cell disorder, nerve conduction studies, and electromyography (EMG) were performed. Multiplex ligation-dependent probe amplification analysis of the SMN1 gene demonstrated a homozygous deletion, confirming the diagnosis of spinal muscular atrophy type I (Tables 2 and 3).

**Table 1:** Creatine phosphokinase (CPK) enzyme levels.

Test name	Results	Reference ranges (IU/L)
CPK	232	Male: up to 190
		Female: up to 165

CPK = creatine phosphokinase.

**Table 2:** Nerve conduction study (NCS) and electromyography (EMG) findings.

**Clinical information provided:** Weakness in bilateral lower limbs. Weak cry. History of myopathy in elder sibling who died at the age of 10 months.
**Plan:** To rule out neuropathy or myopathy or anterior horn cell disorder.
**Conclusion:**
**NCS:** Absent sensory nerve action potential in right median, left ular and bilateral sural nerves. Increased distal motor latency, small compound muscle action potential and reduced motor nerve conduction velocity in right median, left ulnar and right common peroneal nerves. Increased distal motor latency, small compound muscle action potential and normal motor nerve conduction velocity left common peroneal nerves. Small compound muscle action potential, normal distal motor latency, and motor nerve conduction velocity in bilateral tibial nerve.
**EMG:** Severe motor neuropathy affecting the lower extremities more than upper.
**Impression:** The electrophysiological data is consistent with hereditary sensory motor polyneuropathy. A repeat study may be done after 3 to 6 months or may be required to see any progression.

**Table 3:** Genetic testing results of survival motor neuron 1 (SMN1) gene by multiplex ligation-dependent probe amplification (MLPA).

**Test:** SMN1 gene testing by MLPA
**Source:** Blood
**Results:**
Homozygous deletion in *SMN1* gene is detected.
2 copies of *SMN2* gene are present.
**Interpretation:** Absence of SMN1 gene significantly increases the likelihood that this patient is affected with spinal muscular atrophy (SMA). Test results should be interpreted in context of clinical findings, family history and other laboratory data.

SMN2 = survival motor neuron 2.

### 2.2. Management

The patient was managed with supportive care, including nutritional support via a nasogastric tube and respiratory care with monitoring oxygen saturation, chest physiotherapy, and suctioning. Medical management was supervised by a pediatric neurologist. Family support was given by counseling about disease progression and prognosis.

## 3. Discussion

Spinal muscular atrophy, the leading genetic cause of infant mortality, is an autosomal recessive disorder characterized by the degeneration of motor neurons in the spinal cord.^[6,7]^ It is characterized by progressive muscle weakness and atrophy caused by the irreversible loss of anterior horn cells (lower motor neurons) in the spinal cord and brain stem nuclei.^[8]^ In >95% of cases, SMA is caused by a homozygous deletion or mutation of the SMN1 gene, located on chromosome 5q13.^[9]^ SMA type I (Werdnig–Hoffman disease), also known as severe SMA or “non-sitter” SMA, typically presents within the first 6 months of life with hypotonia, areflexia, and poor head control. Affected infants are unable to sit independently and often assume a characteristic frog-leg posture when lying supine. Swallowing difficulties, tongue fasciculations, failure to thrive, and recurrent aspiration are common features of this subtype. SMA type I can be further subdivided into type IA (before birth or within 2 weeks of life), IB (onset < 3 months), and IC (onset 3–6 months).^[1,9,10]^

When spinal muscular atrophy is clinically suspected based on history and physical examination, genetic testing is usually sufficient to confirm the diagnosis.^[9]^ Polymerase chain reaction or multiplex ligation-dependent probe amplification can detect homozygous deletion of exon 7 of the SMN1 gene with approximately 95% sensitivity and nearly 100% specificity.^[11]^ The survival motor neuron 2 (SMN2) gene acts as a modifier gene in SMA, partially compensating for the loss of SMN1 function. Therefore, the number of SMN2 gene copies correlates with disease severity and prognosis. A lower copy number indicates more severe disease, whereas a higher copy number (≥4 copies) is associated with milder phenotypes. Patients with SMA type I commonly have fewer copies of the SMN2 gene, which correlates with earlier disease onset and poorer prognosis.

Additional investigations may be considered when initial genetic testing is inconclusive, including serum CPK levels, nerve conduction studies, EMG, and muscle biopsy. Serum CPK levels are usually normal or mildly elevated; in our case, the level was elevated to 232 IU/L. Nerve conduction studies demonstrated absent sensory action potentials in the right median, left ulnar, and bilateral sural nerves. Needle EMG revealed severe motor neuropathy, predominantly affecting the lower limbs. Muscle biopsy is now largely obsolete due to advances in genetic testing and was not performed in our patient; however, when performed, it typically shows a neurogenic pattern.^[12]^

In our case, management focused on supportive care, including nutritional support, respiratory management, and physiotherapy. Multidisciplinary care was provided by a pediatric neurologist, with an emphasis on symptomatic management and regular clinical monitoring. The parents were counseled about the nature of the disease, its progressive course, expected prognosis and available supportive and therapeutic options. Currently approved disease-modifying therapies by the U.S. Food and Drug Administration include nusinersen, onasemnogene abeparvovec, and risdiplam, all of which increase survival motor neuron protein levels through SMN1 or SMN2-directed pathways.^[13]^

## 4. Conclusion

This case reflects the importance of early identification and timely diagnosis of this rare genetic disorder. Multiple factors like awareness about genetic diseases, diagnostic protocols and availability of cost-effective treatment options like pharmacological drugs and gene therapy are very important to improve outcomes in patients with SMA type I, mainly in resource-limited areas. For a definitive cure, this disease is still a focus for continued research and clinical trials in the medical field.

## Author contributions

**Conceptualization:** Raja Nasir Nawaz.

**Data curation:** Roshaan Bashir.

**Investigation:** Raja Nasir Nawaz.

**Methodology:** Wafa Asjad, Roshaan Bashir.

**Project administration:** Muddassir Khalid.

**Resources:** Wafa Asjad.

**Software:** Sheikh Ali Ahmed Saeed.

**Supervision:** Muddassir Khalid.

**Validation:** Sheikh Ali Ahmed Saeed.

**Visualization:** Muhammad Zain Tayyab.

**Writing – original draft:** Wafa Asjad.

**Writing – review & editing:** Muddassir Khalid.
